# The economic burden of atopic dermatitis in Romania: a broad perspective

**DOI:** 10.3389/fpubh.2024.1531042

**Published:** 2025-01-09

**Authors:** Alin Codruț Nicolescu, Stefan Strilciuc, Rozalina Lăpădatu, Diana-Alecsandra Grad, Cristian Vlădescu, Rodica Olteanu

**Affiliations:** ^1^Emergency Hospital Prof. Dr. Agrippa Ionescu, Bucharest, Romania; ^2^Research Center for Functional Genomics, Biomedicine and Translational Medicine, Iuliu Hațieganu University of Medicine and Pharmacy, Cluj-Napoca, Romania; ^3^RoNeuro Institute for Neurological Research and Diagnostic, Cluj-Napoca, Romania; ^4^Asociația Pacienților cu Afecțiuni Autoimune (APAA), Bucharest, Romania; ^5^National Institute for Management of Health Services, Bucharest, Romania; ^6^Faculty of Medicine, Titu Maiorescu University, Bucharest, Romania; ^7^Dermatology Department, Colentina Clinical Hospital, Bucharest, Romania

**Keywords:** atopic dermatitis, costs, burden, Romania, direct costs, indirect costs

## Abstract

**Introduction:**

Atopic dermatitis (AD), a common dermatological condition, is often associated with significant economic and social burdens. Despite extensive studies globally, there is a gap in understanding the impact of this condition in Romania. This study evaluated the economic burden of AD in Romania, considering both direct and indirect costs.

**Materials and methods:**

A cost of illness study, conducted from a broad perspective, considering 2022 as a reference, using top-down and bottom-up approaches and retrospective and prospective data sources was used to assess direct medical costs (treatments, medical services, hospitalizations), direct non-medical costs (associated costs due to transportation, accommodation), and indirect costs (productivity losses) across four costing scenarios.

**Results:**

In 2022, the total cost of atopic dermatitis in Romania was €29,810,077.2 for adult patients and €133,635,535.2 for pediatric patients, based on a prevalence-based approach, and €5,529,867.8 for adults and €53,175,049.1 for pediatric AD patients when using an incidence-based approach. Medical costs had the highest attributable share of the AD cost for adult patients while productivity costs (inquired by caregivers) had the highest share for pediatric AD patients in both approaches. The overall average annual cost per patient ranged, depending on the scenarios, between €284.72 and €1,045 for adult AD patients and between €293.73 and €9,558.52 for pediatric AD patients.

**Discussion:**

Our results show the increased burden among pediatric AD patients emphasizing the need that future policy interventions should be tailored according the patients’ age.

## 1 Introduction

Atopic dermatitis (AD) is a dermatological disease with a higher attributable burden in terms of disability-adjusted life years/100,000 person-years (99.69 DALYs) than other skin conditions such as contact (28.06 DALYs) or seborrheic dermatitis (3.93 DALYs), or other dermatological diseases such as psoriasis and urticaria (1, 2). AD significantly impacts patients’ lives, affecting their work and personal activities and imposing considerable economic costs (2–4). Studies have reported a high prevalence and impact of AD in specific demographic groups, particularly in children and females. Simpson and colleagues emphasized the prevalence of moderate to severe AD, particularly in younger patients. They highlighted the different severity levels of AD symptoms and their correlation with mental health issues (5). The last Global Burden of Disease estimates shows that Romania had in 2021 the third highest number of prevalent cases (155,583.52; 163,655.1–147,703.89) (6).

Socioeconomic status is an important variable when quantifying the burden of AD, assessing the trends of co-occurring health problems (such as sleep or mental health impairments), and guiding policy efforts for AD patients. A growing body of research highlights AD’s economic and social implications. Findings of a study conducted in Israel reported socioeconomic disparities among AD patients, with those suffering from severe forms often belonging to lower socioeconomic groups and experiencing higher healthcare burdens. In addition, the findings highlight that the direct costs associated with AD were estimated at around $4,411 ($940–$11,536), with indirect costs of $9,068 ($1,289–$15,650), indicating the extensive economic burden of the disease (7). As for productivity lost (due to both absenteeism and presentism), the total number of days summed up to 68.8 (54 days due to presentism and 14.8 due to absenteeism) (8).

AD decreases quality of life, as emphasized by the results of a multinational study across the US and three European countries that outlined differences in AD severity, diagnostic age, treatment approaches, and the correlation between disease severity and quality of life (emphasizing that, regardless of the scale employed to assess QoL, severe patients reported poorer QoL) (6, 7). The broader implications of AD in terms of economic costs are burdening patients and society overall. In their comparative study, Toron and colleagues revealed the heightened economic impact—in UK—of patients with mild-to-moderate AD (as opposed to non-AD patients), including increased healthcare service utilization (for GP visits and dermatology referrals) and associated costs (68.22% higher than that of non-AD patients) (9). In addition, the burden of AD is associated with significant mental health impairments, such as anxiety and depression (5). Increased average annual costs were reported in other European countries: € 5,229 (moderate to severe AD)—Germany, €3,397 (severe AD)—Spain, and €6,993 (uncontrolled AD)—the Netherlands (€6,993) (10–12).

Recently, a study conducted in Romania developed and validated a questionnaire used to estimate national prevalence (13). Despite the emergence of this new national epidemiological study and the international literature on the economic impact of atopic dermatitis, there is a gap in the literature regarding the cost of illness studies in Romania. Therefore, we aimed to provide the first cost of illness study conducted nationally for patients diagnosed with atopic dermatitis. The objectives of this cost of illness study were to provide direct medical, direct nonmedical and indirect costs, as well as to provide utilities (as a secondary analysis) for atopic dermatitis (which afterwards can be incorporated in cost-effectiveness analyses) using EQ-5D-5L data.

## 2 Materials and methods

This cost of illness (CoI) study estimated the economic costs of atopic dermatitis over 12 months (using 2022 as a reference year) from a broad perspective, incorporating both direct and indirect costs and elements of a societal perspective.

### 2.1 Data sources

We used data from several retrospective sources (public and private) and one prospective source (survey data). The list of retrospective sources is the following: Global Burden of Diseases, IQVIA (subsequently referred to as average prescription data), Hospital Consulting (a local financial controlling consultancy firm with national coverage of hospitals), National Institute of Statistics, and National Institute for Health Services and Management.

From IQVIA, we used the average value for medical prescription for patients labeled by IQVIA as “Other dermatitis”—40.4 RON (8.2 €) for 2022. This average was derived from a nationally representative sample of 3,700 pharmacies, taking into account partially or fully subsidized medical prescriptions (thus white prescriptions, which are given for over-the-counter medicine were excluded).

From Hospital Consulting, we used estimates based on financial controlling data, collected from a sample of nationally representative hospitals (in terms of hospital profile and catchment areas). The estimates incorporated in the CoI are the following: average length of hospital stay, for adult patients—7.95 days; and pediatric patients—4.44 days; average cost per hospitalization, for adult patients, 1,094 RON (€219.85) and for pediatric patients, 1,012 RON (€203.37).

From the National Institute of Health Services and Management, we used hospital discharges for which the primary diagnosis was atopic dermatitis listed as “L20.8 (other atopic dermatitis)” 229 hospital discharges and “L20.9 (atopic dermatitis unspecified)”—980 hospital discharges, totaling 1,209.

From the National Institute of Statistics, we used the monthly gross average wage for 2022–6,126 RON (€1231.08), complemented with the number of working days within 2022–251 working days retrieved from an online calculator (14, 15).

From a cross-sectional source, a web survey used to collect data on the usage of health services, costs, and quality of life in patients with AD (that confirmed having a medical diagnosis of AD provided by a doctor), we used responses collected from 622 adult AD patients and 57 pediatric AD patients (for which the data collected was from proxies—caregivers). The survey was constructed using the Surveyworld tool, disseminated on social media by APAA (Asociatia Pacientilor cu Afectiuni Autoimune). The sampling used for the online cohorts was convenience sampling. The start date of the collection process was 25 April 2023 and the end date of the collection process was 18 October 2023. The target participants were patients aged over 18 or their caregivers and the caregivers of patients aged under 18 (children and adolescents) who were diagnosed with atopic dermatitis. In order to access the survey, respondents were required to confirm that the AD diagnosis was put by a physician. If they responded no, they were not able to fill in the survey as they automatically were directed to the ending message. The sample size calculated for this study, aiming for a 95% confidence level and a 4% margin of error, was 600 patients (including responses from caregivers).

For this CoI, we used the following variables: age groups (18–25 years, 26–35 years, 36–45 years, 46–55 years, 56–65 years, 66–75 years, and 75+ years), self-rated AD severity (mild, moderate, severe), employment type (employed full-time, employed part-time, unemployed, retired—age limit, retired—due to sickness, other option, and student) and reported costs for medical services, treatments, associated costs (for transportation, hotel, others) and missing days from work or school due to AD, within the past 12 months. For the question on the number of missing days from work or school, if the responded checked working part-time or full-time, but was in the 18–25 age group, we assume that they listed the number of missing days from work due to AD. We averaged the costs for the three collected cost categories from the survey. However, for pediatric patients, the cost for medical services was not included in the survey, and therefore, for this population segment, we report only costs for treatment services and associated costs (out of the three listed cost categories). The costs for adult and pediatric patients with AD are listed for each severity level. Before computing average costs, missing data for treatment costs for adult patients (type of missing data—Missing Not at Random—MNAR) was imputed using the “mice” (16) R package (after other types of missing data, MCAR)—Missing completely at random and MAR—Missing at random and MCAR—were ruled out using the “naniar” (17) R package, respectively, logistic regression.

We also analyzed the EQ-5D-5L data and report it as a subanalysis. To generate utilities reported by age, sex, and severity—we employed the R package “eq-5d package” (18). In addition, we grouped level 1 under “no problems” and levels 2–5 under “problems.”

From the Global Burden of Disease (GBD) Study 2021 (19) (latest available year), we used crude numbers for Romania, for both sexes, for prevalence <20 years (102,783), prevalence 20+ years (52,801), incidence <20 years (13,125), and incidence 20+ years (8,516) (6). For this CoI, we provide results for both the incidence and prevalence-based. These GBD estimates were also used in calculating the cases corresponding to the three severity levels (for which we have used the total number of active respondents, the active respondents with the specific severity group, and the cases with the specific severity calculated based on the percentage of patients from the survey). Considering that there was an overlap between <20 and 20+ years and the first age group from the survey targeting adult AD patients—18–25 years, we decided to include the number of cases within this age group in the 20+ years segment considering that there are more years covered by this group than the <20 years group.

We calculated the corresponding number of prevalent cases for each of two age groups for each severity level (mild, moderate, and severe cases) based on the corresponding proportion reported in the collected survey data (22.67%—mild, 59.16%—moderate, 18.17% severe, for adult AD patients; 38.6%—mild, 36.84%—moderate, 24.56%—severe, for pediatric AD patients). In addition, we repeated this step when employing the GBD 2021 incidence data. Based on the prevalence and incidence data, we also calculate the incident and prevalent hospitalizations, AD patients and caregivers of AD patients who were full-and part-time workers in order to extrapolate at the national level.

### 2.2 Costing approach

The bottom-up and top-down approaches are employed by the availability of data to yield the total population cost of AD. We used a bottom-up approach to calculate treatment, medical services, and other associated costs (i.e., transportation, accommodation), hybrid approach (combing both bottom-up and top-down approaches) for productivity-related costs, and a top-down approach to calculate hospitalizations.

We evaluated the economic burden of atopic dermatitis (AD) by incorporating both direct medical (hospitalizations, treatments and medical services—for pediatric patients this cost was not included), direct non-medical costs (including associated costs), and indirect costs (productivity losses due to absenteeism). For a subcategory of direct medical costs—treatment costs—we also provide an additional scenario taking into account the average prescription data, thus incorporating a cost comparison for this cost category and evaluating the differences between self-reported costs and retrospective costs that are collected from a nationally representative sample of pharmacies. In addition, we provide costs for adults (the survey data being self-reported) and minors (the survey data being provided by their caregivers). The average for each cost category was multiplied by the number of patients to extrapolate at the national level using GBD prevalence and incidence data disaggregated by severity.

To calculate costs due to hospitalization, we multiplied the average length of stay and average hospitalization costs obtained to the number by patients based on the GBD extrapolations for each severity level (for both prevalence and incidence). These GBD extrapolations are based on the corresponding number for adult and pediatric patients (which were calculated, in the first stage, considering the prevalence sum—for <20 and 20+ years—and the total number of hospitalizations, respectively the incidence sum; in the second stage, the corresponding number for the populations <20 and 20+ years and the percentages corresponding to each severity were used to have the final numbers for the hospitalizations).

To calculate indirect costs related to productivity (for patients aged over 18 years and for caregivers), we incorporated the following elements: age threshold, the number of participants who were considered active in the job market (under the age threshold) either full-time or part-time employed, the gross wage for 2022 (6,126 RON—€1,242.27; for part-time, we used 3,063 RON—€621.13), and the number of working days for 2022 (251 days) (15). The average obtained cost for each employment category and each severity level was then extrapolated using GBD incidence and prevalence. The numbers of full-and part-time workers (used to extrapolate the costs) were calculated using the total number of workers and the number of workers for each severity level from the survey, and the prevalence, respectively, the incidence.

We report average and total AD costs for both incidence and prevalence-based approaches; severity level and descriptive characteristics are reported as totals and percentages.

The Institutional Review Board—Public Health (IRB-PH), Babes-Bolyai University (no. 151122–003) approved the submitted protocol for this cost of illness study.

## 3 Results

Based on the survey severity distribution, there were 22.67% mild cases, 59.16% moderate cases, and 18.17% severe cases for those aged over 20 years and 38.6% mild cases, 36.84% moderate cases, and 24.56% severe cases among those aged under 20 years. Based on the prevalence data, for the 20+ population, there were 11,969 mild patients, 31,239 moderate patients, and 9,593 severe patients while based on the incidence data, there were 1,930 mild patients, 5,038 moderate patients, and 1,547 severe patients. For the <20 years population, there were 39,671 mild patients, 37,867 moderate patients, and 25,245 severe patients based on the prevalence data, while severity distribution based on the incidence data was the following: 5,066 mild patients, 4,836 moderate patients, and 3,224 severe patients.

### 3.1 Results prevalence scenarios

#### 3.1.1 Costs adult AD patients

The total cost of atopic dermatitis in Romania during 2022 for adult patients was €29,810,077.2, of which €17,832,658.32 were attributable to medical costs (€7,862,524.77 to treatment costs, € 9,246,490.26 to costs of medical services, and €723,643.3 to inpatient hospitalization). Direct non-medical costs, represented by associated expenses (transportation, hotel, or other types of expenses) were €6,007,293.39. Indirect costs were €1,432,069.3 due to absenteeism for full-time employed and €4,538,056.2 for part-time employment among AD patients. The yearly average per patient cost for atopic dermatitis in Romania in 2022 for patients aged 20+ years stratified by severity and considering different scenarios can be found in Table 1. In the cost scenario for which we have replaced the collected cost for treatment with the annual average prescription cost, the total cost decreased to €22,380,0128.1 with the total cost corresponding for treatment being €432,575.7.

**Table 1:** Average costs by severity (20+ years).

	Prevalence	Incidence	Prevalence—annual average prescription scenario	Incidence—annual average prescription scenario
Average/all	€564.6	€649.4	€423.9	€508.7
Average/Mild	€284.7	€369.6	€176.6	€261.4
Average/Moderate	€550.3	€635.2	€433.2	€518.1
Average/Severe	€960.1	€1,045	€701.8	€786.7

#### 3.1.2 Costs pediatric AD patients

The total cost of atopic dermatitis in Romania during 2022 for pediatric patients was €133,635,535.2 of which €13,730,848 were attributable to treatment costs, €727,754.2 to inpatient hospitalization (all of these categories corresponding to direct medical costs) and €21,793,718 were attributable to associated expenses (transportation, hotel, or other types of expenses), and €22,081,985.9 due to days off from work for caregivers that reported being full-time employed, and €75,301,229.4 for caregivers that reported being employed part-time (indirect cost). The yearly average cost per patient with atopic dermatitis in Romania in 2022 for patients aged <20 years stratified by severity and considering different scenarios can be found in Table 2.

**Table 2:** Average costs by severity (<20 years).

	Prevalence	Incidence	Prevalence—annual average prescription scenario	Incidence—annual average prescription scenario
Average/total	€1300.2	€4,051.4	€1,174.8	€3,926.03
Average/Mild	€374.9	€758.12	€293.72	€676.9
Average/Moderate	€2,614.8	€9,558.5	€2,491.7	€9,435.4
Average/Severe	€782.2	€967.4	€584	€769.2

In the cost scenario for which we have replaced the collected cost for treatment with the annual average prescription cost, the total cost decreased to €120,746,634.9, with the total corresponding cost for treatment being €842,056.5.

### 3.2 Results incidence scenarios

#### 3.2.1 Costs adult AD patients

The total cost of atopic dermatitis in Romania during 2022 for adult patients was €5,529,867.8 of which €3,598,200.6 were attributable to medical costs (€1,267,955.4 to treatment costs, €1,491,157 to medical services, and €839,088.28 to inpatient hospitalization). Direct non-medical costs, represented by associated expenses (transportation, hotel, or other types of expenses) were €968,773.7. Indirect costs were €230,973.4 due to absenteeism for full-time employed and €731,920.1 for part-time employment. The yearly average per patient cost for atopic dermatitis in Romania in 2022 for patients aged 20+ years is reported in Table 1. In the cost scenario for which we have replaced the collected cost for treatment with the annual average prescription cost, the total cost decreased to €4,331,672.1 with the total corresponding cost for treatment being €69,759.7. Additional costs are reported in Table 3.

**Table 3:** Total direct costs by severity (20+ years).

Prevalence (adult AD patients)			
	Mild (total)	Moderate (total)	Severe (total)
Treatment	€1,392,698.1 (annual average prescription—€98,057)	€3,913,664.59 (annual average prescription—€255,927.6)	€2,556,162.1 (annual average prescription—€78,591.3)
Medical services	€732,513.6	€4,946,243.6	€3,567,733.1
Associated expenses	€852,900.2	€3,051,492.8	€2,102,900.4
Incidence (adult AD patients)			
Treatment	€224,572.5 (annual average prescription—€15,811.7)	€631,167.5 (annual average prescription—€41,274.1)	€412,215.4 (annual average prescription—€12,673.9)
Medical services	€118,117.7	€797,694.4	€575,344.8
Associated expenses	€137,530.6	€492,122.7	€339,120.9

#### 3.2.2 Costs pediatric AD patients

The total cost of atopic dermatitis in Romania during 2022 for pediatric patients was €53,175,049.1, of which €1,753,842.2 were attributable to treatment costs, €668,113.6 to inpatient hospitalization (all of these categories corresponding to direct medical costs) and €2,783,027 were attributable to associated expenses (transportation, hotel, or other types of expenses, which is a direct non-medical cost), and €9,507,797.7 due to days off from work for caregivers that reported being full-time employed, and €38,462,727.7 for caregivers employed part-time (indirect costs). The yearly average per patient cost for atopic dermatitis in Romania in 2022 for patients aged <20 stratified across severity levels and scenarios is reported in Table 2. In the cost scenario for which we have replaced the collected cost for treatment with the annual average prescription cost, the total cost decreased to €51,529,194.2 with the total corresponding cost for treatment being €107,527.4. Additional costs are reported in Table 4.

**Table 4:** Total direct costs by severity (<20 years).

Prevalence (pediatric patients)			
	Mild (total)	Moderate (total)	Severe (total)
Treatment	€3,547,085.3 (annual average prescription—€325,007.3)	€4,973,006.9 (annual average prescription—€310,227.9)	€5,210,755.8 (annual average prescription—€206,821.3)
Medical services	€0	€0	€0
Associated expenses	€4,314,982.7	€6,691,554.2	€10,787,181
Incidence (pediatric patients)			
Treatment	€452,952.6 (annual average prescription—€41,503.54)	€634,972.1 (annual average prescription—€39,611.1)	€665,457.6 (annual average prescription—€26,412.83)
Medical services	€0	€0	€0
Associated expenses	€551,010.84	€854,402.6	€1,377,614.2

### 3.3 Productivity costs

#### 3.3.1 Productivity adult AD patients

Based on the results derived from the GBD prevalence, among the full-time workers, there were 2,163 mild AD patients, 17,125 moderate AD patients, and 2,600 severe AD cases, while for part-time workers, there were 1,496 mild patients, 21,477 moderate patients, and 1799 severe AD patients. Based on the results derived from the GBD incidence, among full-time workers, there were 349 mild AD patients, 2,762 moderate AD patients, and 419 severe AD patients while for part-time workers, there were 241 mild AD patients, 3,464 moderate AD patients, and 290 severe AD patients. Productivity costs were highest among patients with moderate AD, for both full-and part-time employment (for both prevalence and incidence-based analysis).

#### 3.3.2 Productivity caregivers pediatric AD patients

Based on the results derived from the GBD prevalence, there were 13,224 mild AD patients, 16,830 moderate AD patients, and 5,610 severe AD patients that had caregivers that had productivity losses while being employed full-time, and 37,867 moderate AD patients for which caregivers were employed part-time. As for the results derived from the incidence, there were 1,689 mild AD patients, 2,149 moderate AD patients, and 716 severe AD patients that had caregivers that had lost productivity costs while being employed full-time, and 4,836 that had caregivers that had productivity losses while being employed part-time. Productivity costs for both adult and caregivers of pediatric patients are reported in Table 5.

**Table 5:** Productivity costs by severity.

Productivity adult patients with AD				
	Prevalence		Incidence	
	Full-time	Part-time	Full-time	Part-time
Mild	€107,056.4	€158,673	€17,267	€25,592.4
Moderate	€918,088.9	€3,934,310	€148,073.8	€634,543.4
Severe	€406,923	€445,073	€65,632.5	€71,784.3
Productivity caregivers of pediatric patients with AD				
Mild	€6,731,690.9	€0	€2,578,839.7	€0
Moderate	€11,780,459	€75,301,229.4	€6,017,216.2	€38,462,727.7
Severe	€3,569,836.1	€0	€911,741.8	€0

### 3.4 Hospitalization costs

#### 3.4.1 Hospitalizations adult AD patients

There were 93 hospitalizations among mild AD patients, 243 among moderate AD patients, and 75 among severe adult AD patients, considering prevalence-based estimations. However, when considering incidence-based estimations, there were 108 mild AD patients, 281 moderate AD patients, and 86 severe AD patients.

#### 3.4.2 Hospitalizations pediatric AD patients

Regarding the pediatric population, there were 308 among mild AD patients, 294 among moderate AD patients, and 196 among severe AD pediatric patients, considering prevalence-based estimations. However, when considering incidence-based estimations, there were 283 mild AD patients, 270 moderate AD patients, and 180 severe AD pediatric patients. Hospitalization costs reported across severity and population segments are reported in Table 6.

**Table 6:** Hospitalization costs by severity (20+ years and < 20 years).

Severity	Adult AD patients—prevalence	Adults AD patients—incidence	Pediatric AD patients—prevalence	Pediatric AD patients—incidence
Mild	€164,041.3	€190,211.3	€280,887.6	€257,868.4
Moderate	€428,136.2	€496,438.1	€268,120	€246,147
Severe	€131,465.7	€152,438.9	€178,746.64	€164,098.1

### 3.5 Results sub-analysis EQ-5D-5L

The utilities for mild cases range between 0.017 and 1, moderate cases between 0.136 and 1, and severe cases between 0.483 and 0.947 (Table 7). The utilities for female patients (adult segment) ranged between 0.017 and 1, while for male patients, between 0.581 and 1. As for the pediatric population, utility values ranged between 0.349 and 1 for females and-0.025 and 1 for male patients. Descriptive statistics for EQ-5D-5L can be found in Tables 8–11 for both adult and pediatric patients.

**Table 7:** Input cost averages by categories and 20+ and < 20.

	20+ population	<20 population	20+ population	<20 population	20+ population	<20 population
Cost	Mild AD		Moderate AD		Severe AD	
Treatment cost	€116.4	€89	€125.3	€131	€266.5	€206.4
Medical services cost	€61.2	€0	€158.3	€0	€371.9	€0
Associated cost	€71.3	€109	€97.7	€177	€219.2	€427.3

**Table 8:** Frequencies of issues reported by AD patients in Romania by EuroQol dimension and age group.

EQ-5D Dimension	18–25 years	26–35 years	36–45 years	46–55 years	56–65 years	66–75 years	75+ years
Mobility—no problems	82.8%	90.6%	88.8%	80.5%	88.9%	83.1%	88.9%
Mobility—problems	17.2%	9.4%	11.2%	19.5%	11.1%	16.9%	11.1%
Self-care—no problems	55.2%	31.3%	35.7%	32%	36.5%	32.5%	55.6%
Self-care—problems	44.8%	68.8%	64.3%	68%	63.5%	67.5%	44.4%
Usual activity—no problems	82.8%	90.6%	87.4%	80.5%	85.7%	83.1%	66.7%
Usual activity—problems	17.2%	9.4%	12.6%	19.5%	14.3%	16.9%	33.3%
Pain/discomfort—no problems	37.9%	34.4%	31.5%	26%	39.7%	24.1%	33.3%
Pain/discomfort—problems	62.1%	65.6%	68.5%	74%	60.3%	75.9%	66.7%
Anxiety/depression—no problems	27.6%	21.9%	26.6%	23.5%	34.9%	33.7%	55.6%
Anxiety/depression—problems	72.4%	78.1%	73.4%	76.5%	65.1%	66.3%	44.4%

**Table 9:** EQ-VAS collected from AD patients in Romania.

EQ VAS	18–25 years	26–35 years	36–45 years	46–55 years	56–65 years	66–75 years	75+ years
Mean—Std Dev	67.24 (24.98)	70.56 (22.30)	66.26 (22.12)	67 (21.13)	66.80 (20.16)	59.09 (21.54)	65.33 (14.65)
Median	70	72.5	70	70	70	65	60
25th percentile	55	53.75	50.5	53.75	55	40	53
75th percentile	85	90	82.5	85	80	75	70

**Table 10:** Frequencies of issues reported by caregivers of pediatric AD patients in Romania by EuroQol dimension and age group.

EQ-5D Dimension	1–10 years	10–17 years
Mobility—no problems	91.66%	80.25%
Mobility—problems	8.33%	19.05%
Self-care—no problems	47.22%	42.86%
Self-care—problems	52.78%	57.14%
Usual activity—no problems	69.44%	66.66%
Usual activity—problems	30.55%	33.33%
Pain/discomfort—no problems	44.44%	55.55%
Pain/discomfort—problems	55.55%	44.44%
Anxiety/depression—no problems	44.44%	19.05%
Anxiety/depression—problems	55.55%	80.95%

**Table 11:** EQ-VAS collected from AD patients in Romania.

EQ VAS	1–10 years	11–17 years
Mean—Std Dev	76.81 (19.97)	66.9 (23.53)
Median	80	65
25th percentile	65	50
75th percentile	91.25	90

## 4 Discussion

This is the first cost of illness measuring the economic burden of AD among adult and pediatric AD patients in Romania. Our results highlight that the highest cost (per patient) for adult AD patients is for moderate and severe cases across all four scenarios, with differences for mild cases being between 1.72 and 2.45 times lower compared to moderate AD cases. Regarding pediatric AD patients, the highest cost (per patient) is among moderate AD, for all scenarios, with the average cost per mild patients being between 7 and 12.6 times lower (compared to the cost for average cost per moderate AD case) and between 2.83 and 4 times lower compared to average costs for severe cases. Regarding total direct costs, for both incidence and prevalence approaches, the highest costs for adult AD patients were among moderate AD patients, for medical services. Regarding indirect costs, the highest costs were obtained for moderate AD patients working part-time and caregivers of moderate AD pediatric patients that were working part-time (based on the prevalence-based approach). As for pediatric AD, the highest costs, considering both incidence and prevalence approaches, were for associated expenses, for severe patients. As for hospitalizations, the highest totals for adult AD patients were for moderate patients (for both incidence and prevalence) while for AD pediatric the highest is for mild patients.

In a study conducted in Germany between 2017 and 2019, the annual costs per patient considering severity was highest among patients with moderate-to-severe AD (€ 5,229 ± € 7,538) compared to patients with mild AD (€ 1,466 ± € 3,029). The total annual costs for all patients were estimated to be more than € 2.2 billion, a figure significantly higher than that in Romania (10). This difference could be attributed to various factors, including differences in healthcare systems, cost of living, in addition to differences in study methodology. A European Union-5 study that employed 2017 data for France, Germany, Italy, Spain, and the UK quantified the economic burden considering disease severity according to the Dermatology Life Quality Index (DLQI) score. The highest costs per patient (for EU-5) were reported for the last DLQI category „extremely large” (€6,924—average annual direct costs, €14,236—average annual indirect costs). The countries with the highest direct costs for “extremely large” DLQI severity category were Germany (€9,894), UK (€7,055), and France (€2,764). As for the moderate DLQI severity category, the highest costs were reported in the UK (€2,397), France (€2,764) as well as Germany (€3,028) (20). This study also points out an increased burden corresponding to indirect costs, with Germany and the UK having the highest costs for both the “extremely large” DLQI severity category (Germany, €13,813; the UK, €16,150), respectively moderate DLQI severity category (Germany, €8,655, the UK, €7,685) (20).

Our study highlights that the sum two out-of-pocket (OOP’s) categories—treatment and medical services—represent the highest attributable share of direct medical costs for adult population. However, when comparing the costs inquired for treatments with average prescription costs (based on partial or fully reimbursed treatment) vs. costs collected from patients via web survey (for both adult and pediatric population) emphasizes the cost inequalities as most treatments are not reimbursed. In addition, our findings report the increased burden attributable to associated costs (transportation, hotel, etc.), direct non-medical costs, as this points out inequalities regarding the distribution of healthcare providers (21, 22).

Among pediatric AD patient, although the highest economic burden attributable to treatment was among severe patients, while in the IQVIA scenario, the highest t economic burden attributable to treatment was among mild patients. This was due to the fact that, although a higher average cost was reported in the survey, most prevalent cases were mild while least were severe.

A global survey conducted by the National Eczema Association on OOPs reported median cost per year, per patient of $600 (however, for 42% of AD patients reported costs higher than $1,000 due to OOPs) (23). Productivity costs were the highest, for part-time workers, for adult AD patients and caregivers of pediatric AD patients. However, our study measured these costs considering only productivity costs due to absenteeism. In a sample composed of patients with AD from Europe (France, Germany, and the UK) and the US diagnosed with moderate-to-severe AD, impairment in work productivity (measured using absenteeism) was higher in the patients with severe AD. The number of working days lost due to absenteeism per study sample was 0.5 days/week (24). Another study focusing on patients with AD living in Japan showed that the percentage of time missed from work per day in the group with mild AD was slightly higher than the percentage reported by the moderate/severe AD group; however, the difference was not statistically significant (25). Another cost of illness study, conducted in Hungary and focusing only on adult patients with AD, highlighted the considerable costs due to productivity losses (for both absenteeism and presentism). These findings align with our study’s relevation of the substantial indirect costs, including transportation and accommodation for medical appointments in Romania. However, the Hungarian study’s use of the Work Productivity and Activity Impairment (WPAI) questionnaire provides a more quantified view of the impact of AD on work productivity. While both our and the Hungarian study provide data on the high cost of managing AD, the Hungarian study’s detailed analysis of the relationship between disease severity, duration, quality of life, and economic burden offers a more detailed analysis of the relationship between disease severity, duration, quality of life, and economic burden (26). The economic burden of atopic dermatitis among pediatric AD patients is high as well. In Singapore, the average annual cost for mild $6,651, while the cost for severe cases was 2.16 times higher (with the highest share being due to informal care and the lowest due to healthcare services) (27). In Italy, the average annual cost is highest among severe patients ($2,224) and lowest among mild patients ($853) (28). In Sweden, the difference among mild-to-moderate and severe patients for average medical cost was of €795 (€1906—severe AD patients) (29).

### 4.1 Strengths

To our knowledge, this is the first study that reports the cost of illness of AD in pediatric and adult populations and provides data on the quality of life on AD in Romania. Most studies assessed productivity-related costs only for full-time employees. However, our study includes part-time workers in order to provide a more complex cost assessment considering different working arrangements. Our study included direct non-medical costs in addition to only having medical costs for the direct cost category.

### 4.2 Limitations

Nevertheless, our study has some limitations. The survey used that collected several of the included variables in our cost of illness study use convenience sampling (which may not fully represent the entire population affected by AD and could introduce bias by disproportionately representing specific demographics) and collecting the data using self-reported questionnaire (which may be valuable for gathering personal experiences but subjected to recall and social desirability biases as respondents may not accurately remember or selectively report their expenditures and quality of health related to AD). Another limitation (related to data availability and methodological considerations), to a certain extent, was using both top-down and bottom-up approaches as the survey data covered only several items, we computed the other items based other available data sources (this method was employed in other cost of illness studies considering data availability constraints and being recommended in case of data fragmentation) (30–33). In addition, we clearly indicate in the methodology and results section the obtained results for the treatment cost considering both scenarios—average obtained from the survey and the annual average prescription cost. This mixed methodology has some challenges regarding data harmonization, but it allowed to capture costs that would have been missed using solely one approach. Future cost of illness studies should try to cover as many cost items as possible, and have a shorter recall period (less 12 months, which was used for the survey). Given the study’s focus on Romania, the findings might not be generalizable to other countries or regions with different healthcare systems, economic conditions, and demographic compositions; however, considering that we extrapolated the costs using nationally representative data from the GBD 2021 study, our findings are representative for Romania. Our study did not include clinical data such as disease severity assessed by healthcare professionals (the variable disease severity being self-reported and collected using our survey), which could provide a more comprehensive view of the disease burden. In addition, when quantifying productivity losses, we focused only on absenteeism and have not included presentism. In addition, we do not have data in regards to controlled or uncontrolled AD, as this aspect could greatly influence the economic burden and quality of life.

## 5 Conclusion

The substantial economic burden and highlighted in this study underscore the need for targeted healthcare policies and interventions in Romania. These should aim at reducing the direct and indirect costs of AD, improving access to effective treatments, addressing the associated mental health issues, and considering the integration of effective management in public policies (34).

Understanding the concept of endotypes in AD provides a framework to rationalize treatment costs based on scientific evidence, moving beyond the current practice of determining treatment primarily by disease presence and severity. This approach enables a more personalized management strategy that considers the heterogeneity of the disease and associated comorbid conditions, such as asthma, nasal polyposis, and allergic conjunctivitis. Recent insights into endotypes suggest that aligning therapeutic interventions with distinct pathophysiological pathways may not only optimize patient outcomes, but also justify resource allocation by targeting interventions more effectively. This paradigm shift emphasizes the need for integrating comorbidities into treatment planning to enhance both clinical and economic efficiency in managing AD (35, 36).

Integrating our findings into healthcare planning and resource allocation could lead to more effective management of AD, ultimately enhancing patient outcomes and reducing the societal burden of this condition. Future research should focus on quantifying the impact of atopic dermatitis by employing a longitudinal approach (with 2 or more data collection timepoints) including more specific out-of-pocket cost categories, healthcare utilization, and quality of life across a single sample. In addition, data collection efforts should shift to face-to-face data collection, in inpatient or outpatient settings and should be facilitated by medical professionals. In conclusion, this study provides results for understanding of AD’s economic and social impact in Romania, aiming to educate policymakers on the burden of AD. The insights from this research could serve as a foundation for future studies and inform healthcare strategies to mitigate the multifaceted challenges posed by atopic dermatitis.

## Data Availability

The raw data supporting the conclusions of this article will be made available by the authors, without undue reservation.
